# The Emerging Immunological Role of Post-Translational Modifications by Reactive Nitrogen Species in Cancer Microenvironment

**DOI:** 10.3389/fimmu.2014.00069

**Published:** 2014-02-24

**Authors:** Francesco De Sanctis, Sara Sandri, Giovanna Ferrarini, Irene Pagliarello, Silvia Sartoris, Stefano Ugel, Ilaria Marigo, Barbara Molon, Vincenzo Bronte

**Affiliations:** ^1^Immunology Section, Department of Pathology and Diagnostics, University of Verona, Verona, Italy; ^2^Istituto Oncologico Veneto, Istituto Di Ricovero e Cura a Carattere Scientifico, Padua, Italy; ^3^Venetian Institute of Molecular Medicine, Padua, Italy

**Keywords:** RNS, nitrotyrosine, cancer, microenvironment, immune escape

## Abstract

Under many inflammatory contexts, such as tumor progression, systemic and peripheral immune response is tailored by reactive nitrogen species (RNS)-dependent post-translational modifications, suggesting a biological function for these chemical alterations. RNS modify both soluble factors and receptors essential to induce and maintain a tumor-specific immune response, creating a “chemical barrier” that impairs effector T cell infiltration and functionality in tumor microenvironment and supports the escape phase of cancer. RNS generation during tumor growth mainly depends on nitric oxide production by both tumor cells and tumor-infiltrating myeloid cells that constitutively activate essential metabolic pathways of l-arginine catabolism. This review provides an overview of the potential immunological and biological role of RNS-induced modifications and addresses new approaches targeting RNS either in search of novel biomarkers or to improve anti-cancer treatment.

## Introduction

During tumor progression, cancer cells secrete factors, like cytokines, chemokines, and metabolites (1, 2) that promote the development of a flexible microenvironment able to induce a modification in anti-tumor immune response. Tumors, indeed, need to shade and block out the effectors of the immune system in order to keep on growing (3). For this purpose, neoplastic cells release soluble factors that alter the physiological hematopoiesis in bone marrow and secondary lymphoid organs, such as the spleen, by promoting an unusual commitment of highly immunosuppressive immature myeloid cells that sustain tumor growth, neovascularisation, and spreading to local and distant anatomical sites (4, 5). These cells, which have been named myeloid-derived suppressor cells (MDSCs) to highlight their common myeloid origin and immunoregulatory properties on tumor-specific effector T lymphocytes, critically contribute to shape an immune privileged environment (6). MDSCs represent a heterogeneous population, which includes immature monocyte- and granulocyte-like elements, and progressively engrafts the tumor and secondary lymphoid organs in many preclinical contexts, such as in transgenic mouse models of autochthonous pancreatic adenocarcinoma (7). Expansion of MDSCs has been described also in clinical studies demonstrating an inverse correlation between MDSC levels and cancer clinical stage and outcome (8, 9). The other predominant and heterogeneous myeloid cell subset induced by tumor progression is represented by tumor-associated macrophages (TAMs), which are classified in two distinct subgroups: classically activated, type 1 macrophages (M1) and alternatively activated, type 2 macrophages (M2) (10–14). In many solid cancers, tumor-infiltrating macrophages are polarized toward an M2-phenothype and promote tumor progression by sustaining tumor angiogenesis and providing essential growth factors. In agreement with their pro-tumoral activity, the M2-like macrophage presence and location within tumor is often correlated to a poor prognosis (15–17). Tumor-derived soluble factors and tumor-associated myeloid cells build up a “physical barrier” around the tumor cells, which protects them against the attack of cytotoxic T lymphocytes (CTL) and natural killer (NK) cells (18). Moreover, both neoplastic and tumor-associated myeloid cell metabolisms are responsible for the consumption of essential nutrients required to sustain lymphocytic activity, such as l-arginine (l-Arg), l-cysteine (l-Cys), l-phenylalanine (l-Phe), and l-tryptophan (l-Trp) (5, 19): depletion of these amino acids establishes an additional “chemical barrier” around the tumor that affects T cell fitness (20–23). One of the most essential component of this barrier is represented by the diatomic radical nitric oxide (nitrogen monoxide, NO), which is involved in a large spectrum of immune dysfunctions such as T cell inhibition through the blockade of phosphorylation and activation of Janus kinase (JAK) 3, reduction of major histocompatibility complex class II (MHCII) expression, and induction of T cell apoptosis (24). NO is endogenously generated by four isoforms of nitric oxide synthase (NOS): mitochondrial-specific NOS (mtNOS), neuronal NOS (nNOS or NOS1), inducible NOS (iNOS or NOS2), and endothelial NOS (eNOS or NOS3). The mtNOS, nNOS, and eNOS are constitutively expressed in a variety of cell types and are activated following a transient change in intracellular calcium concentration. On the contrary, iNOS is expressed in response to several extracellular stimuli regulating distinct signaling pathways and catalyzes NO production independently from intracellular calcium concentration (25). Interestingly, iNOS enzyme is expressed by M1-like macrophages and can be co-expressed with arginase-1 (ARG1) in MDSCs, resulting in l-Arg reduced availability and production of reactive nitrogen species (RNS) in tumor microenvironment. RNS affect not only the structure of individual proteins but also modify the protein–protein interaction and function (26). Thus, nitrogen-derived post-translational modifications (PTM) result from either direct interaction with NO or after exposure to RNS, a term that includes peroxynitrite (ONOO^−^), nitrogen dioxide radical (NO_2_), and other nitrogen oxides and products arising when NO reacts with reactive oxygen species (ROS) such as superoxide anion (O_2_^−^) (27). RNS are commonly generated in sub-cellular compartments such as the peroxidase-containing secretory granules of neutrophils and eosinophils, the mitochondria and endoplasmic reticulum of endothelial and smooth muscle cells, and the vasculature (28). Under physiological conditions, cells produce ROS through the mitochondrial electron transport of aerobic respiration. At low levels, ROS are well tolerated by cells thanks to enzymatic detoxifying systems like superoxide dismutases (SOD) and glutathione *S*-transferase, which neutralize the free radicals (29). Under pathological contexts (such as during sustained inflammation), ROS reach high concentrations and become toxic, thus affecting cellular metabolism by either direct oxidations of macromolecules or after combining with NO to generate RNS. The reaction between NO and O_2_^−^ is extremely rapid and depends on both redox environment and NO concentrations, producing two distinct PTM: nitration or nitrosylation. An equal concentrations of NO and superoxide leads to peroxynitrite formation, which can nitrate either free or protein-associated tyrosines to form 3-nitrotyrosines; two- to three-fold excess of NO predominantly results in dinitrogen trioxide (N_2_O_3_) formation, the major *S*-nitrosylating agent in the intracellular microenvironment (30).

**Figure 1:**
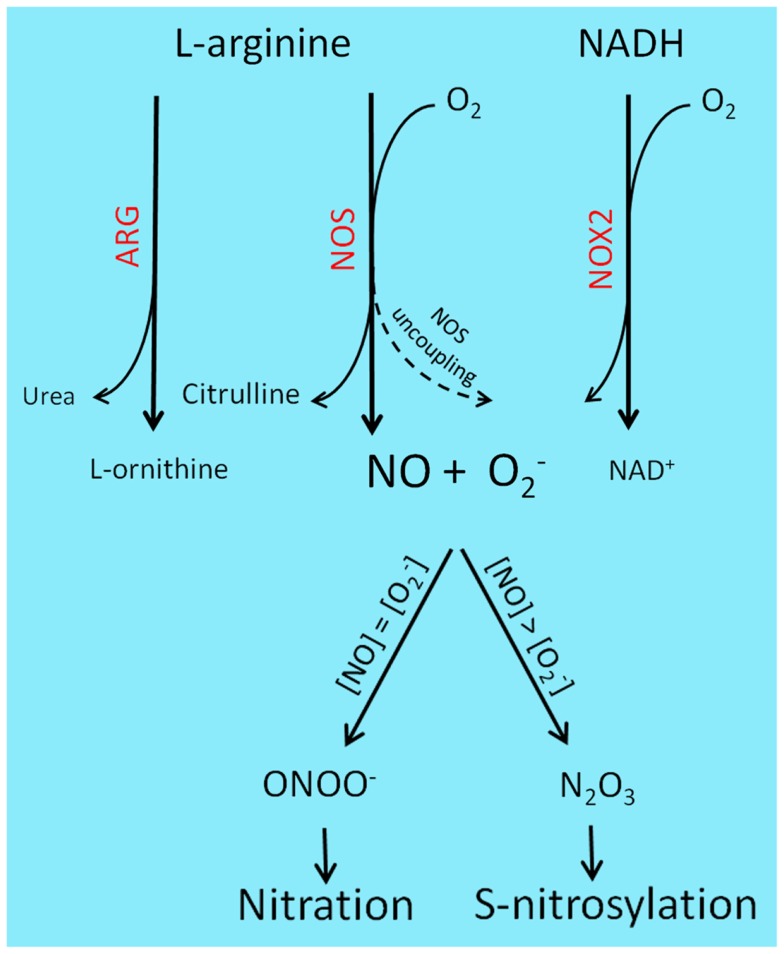
**Reactive nitrogen species-generating chemistry and PTM**. l-Arginine catabolism involves two main classes of enzymes, ARG and NOS. ARG participates in the urea cycle, producing l-ornithine and urea, while NOS generates nitric oxide and citrulline. l-Arginine deprivation by ARG up-regulation results in NOS uncoupling and consequent production of ${\text{O}}_{2}^{-}$ rather than NO. Superoxide is also produced by NOX enzyme, during the transfer of electrons from NAD(P)H to molecular oxygen. Under pathological contexts, such as cancer and sustained inflammation, NO and ${\text{O}}_{2}^{-}$ levels raise and combine to generate a variety of RNS: equal concentrations of NO and superoxide will lead to ONOO^−^ formation and subsequent protein nitration, while two- to three-fold excess of NO will predominantly result in N_2_O_3_ production, a main *S*-nitrosylating agent.

A representation of the main RNS-generating pathways and derived PTM is depicted in Figure 1.

Protein tyrosine nitration consists in the incorporation of a nitro triatomic molecule (−NO_2_), generally in *ortho* position of the phenol hydroxyl group. There are two main mechanisms leading to tyrosine nitration *in vivo*: the formation of peroxynitrite and the production of NO_2_ by heme proteins (31). Peroxynitrite chemistry is highly pH dependent. At physiological pH, NO reacts with O_2_^−^, producing ONOO^−^. Nitration is maximal at physiological pH (about pH 7.4) and its yield decreases quickly under more acidic or basic conditions, whereas sulfhydryl oxidation increases from pH 5 to 9 (32). Peroxynitrite-mediated tyrosine nitration is also accelerated in the presence of transition metal ions either in their free form (Cu_2_^+^, Fe_3_^+^, Fe_2_^+^), as complexes involving protoporphyrin IX (hemin), chelators (cyanide (CN^−^), or ethylenediaminetetraacetic acid (EDTA)) (33).

Nitration is commonly considered an irreversible modification that can be removed only with protein turnover. However, some papers describe the presence of denitrases in different tissues and animal models making controversial this assumption: for example, a denitrase activity was detected in rat and dog tissues (34–36). Moreover, this denitrating activity was described in lipopolysaccharide (LPS)-activated macrophage lines (37, 38). Interestingly a nitration reversible/enzymatic mechanism active in the mitochondria after changes in O_2_ concentrations was also identified (39). Since RNS-dependent PTM have critical effects on protein function, the reversibility of the process may unveil a crucial biological regulation mechanism for cellular metabolism. The frequency of tyrosine occurrence in proteins is approximately 3–4 mol%, which is similar to other aromatic amino acid such as phenylalanine (40). Tyrosine is a mildly hydrophobic amino acid and so there is a relatively good probability that some tyrosine residues will be exposed to the solvent. Examination of the nitration site in peroxynitrite-modified proteins revealed a preference for tyrosine residues located in loop structures and paucity of nitration in tyrosine residues located on β-strands; however, the primary protein structure does not seem to be a discriminative factor since no consensus sequence for tyrosine nitration has been found so far (40). The addition of a nitro group to tyrosine confers particular physicochemical properties to the modified amino acid, which may have important functional consequences. Three major effects on protein function can be predicted due to tyrosine nitration: no alteration in protein function, loss of function, and gain of function. For example, the plasma proteins α1-antichymotrypsin and transferrin do not undergo any substantial function modification following tyrosine nitration, either *in vitro* or *in vivo* (41); on the contrary specific enzymes, as glutathione reductase (GR) and protein kinase C, are inactivated by 3-nytrotyrosine formation (42). On the other hand, cytochrome c (43), fibrinogen (44), protein kinase Cε (45), and microsomal glutathione *S*-transferase 1 (46) present an increased functionality following PTM.

In addition to nitration, NO could induce other important modifications including protein S-nitrosylation. This is a covalent PTM resulting from the coupling of a NO moiety with the reactive thiol group of a cysteine residue to form an *S*-nitrosothiol. Contrary to other PTM, such as phosphorylation or ubiquitinylation, S-nitrosylation is not enzyme-dependent. Protein S-nitrosylation is reversibly controlled by S-nitrosylation and denitrosylation reactions, which preserve a dynamic balance in living systems. A number of studies have indicated that abnormal S-nitrosylation is implicated in cancer development and progression as in head and neck squamous cell carcinoma (47), as well as in response to some therapeutic and treatment options (48). Indeed, several *S*-nitrosylated proteins have been identified to be involved in various cancer-related events. Briefly, the B cell lymphoma (Bcl)-2 S-nitrosylation, which occurs in Cys_158_ and Cys_229_ residues, reduces its degradation through the ubiquitin–proteasomal pathway exacerbating the BCL-2 anti-apoptotic effect (49); the S-nitrosylation of apoptosis antigen 1 (Fas) on its Cys_304_ promotes Fas ligand-mediated apoptosis in cancer cells (50); the S-nitrosylation of p53 prevents normal response to damaged DNA (51); the S-nitrosylation of hypoxia inducible factor (HIF)-1α exerts a pro-angiogenesis function in tumor progression and confers resistance to radiotherapy (52); finally, the S-nitrosylation of *O*^6^-alkylguanine-DNA alkyltransferase (AGT) results in a catastrophic and irreversible failure of DNA repair system, suggesting that S-nitrosylation may contribute to cancer development through its indirect role in preventing routine DNA repair in otherwise normal cellular systems (53). RNS-induced PTM may affect important physiological processes such as apoptosis and autophagy that promote tumor onset and neoplastic evolution. Autophagy is a cellular process of turnover for useless proteins and organelles, which is exploited by tumor cells to survive under conditions of metabolic stress, such as oxidizing environments and nutrient starvation (54). Through autophagy cells may survive for a while, by progressively shrinking in size and reducing their metabolism; however, they eventually die if better conditions do not occur; thus autophagy can be considered a temporary and reversible path to cell death. It has been shown that RNS induces autophagy through suppression of mTORC1 pathway decreasing cell viability in MCF-7 breast cancer cell line (55).

All these modifications can also modulate immunological responses and impair any immune-based cancer therapeutic approach; for this reason, in this review, we will focus on the generation, the biological role and the possible strategies to identify proteins subjected to PTM and nitrosative stress in order to design new diagnostic tools and target RNS in tumor microenvironment.

## Factors Promoting RNS Generation in Tumor Microenvironment during Cancer Progression

Reactive nitrogen species generation relies on the expression and activity of three main enzymes: NADPH oxidase (NOX), NOS, and ARG. Although contribution of malignant cell to peroxynitrite production is possible, most of the nitrosative stress is considered a reflection of the activation of these specific enzymes in tumor-infiltrating myeloid cells, such as TAMs and MDSCs.

### NADPH oxidase 2

NADPH oxidase 2 (NOX2) is mainly expressed in leukocytes and endothelial cells (56) and it is activated via interaction of vascular endothelial growth factor (VEGF) with VEGFR, tumor necrosis factor (TNF)-α with TNFR1, and angiopoietin with tyrosine kinase with immunoglobulin and epidermal growth factor (EGF) homology domains (Tie) 2 (57). Signal transducer and activator of transcription STAT3 directly regulates the expression of both regulatory (p47phox) and catalytic (gp91phox) NOX2 subunits (58) and controls the synthesis of S100A8 and S100A9, which potentiate NOX2 activity (59). Similarly, IFN-γ up-regulates NOX2 via JAK/STAT1 pathway (60). Interestingly, the protein complex activity is finely tuned by other post-translational mechanisms, such as phosphorylation patterns and subunits availability (61).

### iNOS/NOS2

iNOS/NOS2 expression is mainly regulated at the transcription level and the nuclear factor kappa-light-chain-enhancer of activated B cells (NF-κB) represents the central switch. In response to specific signals, NF-κB is released from IκB and it can translocate to the nucleus, promoting the transcription of downstream genes (62). As demonstrated by mutational analysis, NF-κB acts through multiple binding sites on *iNos* promoter, suggesting a modulation of the response proportional to its availability, which in turn is regulated by immune stimulatory signals such as LPS, IL-1β, TNF-α, IFN-γ, and oxidative stress (63). Conversely, TGF-β, antioxidants and glucocorticoids inhibit NF-κB and, consequently, iNOS synthesis (64). *iNos* promoter comprises binding sites for other transcription factors including CCAAT/enhancer-binding protein (c/EBP), activation protein (AP)-1, interferon regulatory factor (IRF)-1, p53, STAT3, and octamer factors (65–69). Interestingly, cellular iNOS concentration is finely tuned also at a translational and post-translational level by either loss of stabilizing proteins at 3′ untranslated region of *iNos* mRNA (70, 71) or increase in iNOS turnover in response to caveolin-1, TGF-β, peroxisome proliferator-activated receptor (PPAR)-α agonist, and calpain (72–77).

### Arginases

Arginases exist as two isoenzymes in mammals, ARG1 and ARG2 differing for cellular expression/localization and for immunological properties (78). ARG1 takes part in the urea cycle in the liver and it is localized in the cytoplasm, whereas ARG2 is a mitochondrial protein mostly present in kidney, prostate, and small intestine, where the urea cycle is not complete. STAT6 and c/EBPβ are the main transcription factors controlling arginases in myeloid cells (79). ARG1 expression can be regulated by the cytokines IL-4, IL-10, and IL-13 inducing the interaction of PU.1, STAT 6, and c/EBPβ transcription factors on the enhancer element of *Arg1* promoter (80). Mouse *Arg1* is also regulated by COX2-dependent PGE2 overproduction (81) and by macrophage stimulating protein (MSP) (82). At a post-translational level, ARG1 is stabilized by S-nitrosylation on Cys_303_ residue, which can depend on an increased iNOS activity (83). All the molecular pathways listed above participate in regulating ROS and RNS-producing enzymes. As further discussed below, cancer exploits these mechanisms pushing the balance toward the expression of NOX, iNOS, and ARG1 with the purpose of increasing ROS and RNS levels tailoring a microenvironment that promotes immune privileged conditions and can sustain its unrestricted growth.

One of the strategies developed by neoplastic cells in tuning the immune system is based on the release of factors that promote the expression of RNS-generating molecules, i.e., HIF1-α, which rises after exposure to hypoxic environments. The synthesis of angiogenic factors (such as VEGF) is promoted by HIF1-α, a transcription factor that is usually quickly degraded in the proteasome after the hydroxylation carried out by the poly hydroxyl domain (PHD) proteins under normal oxygen conditions (84). Current data underline how HIF1-α may trigger broader effects than those related to angiogenesis and support tumor growth also by promoting local immune suppression. The hypoxic tumor microenvironment is indeed able to modify the phenotype of MDSCs by increasing their immunosuppressive properties and promoting their differentiation to TAMs. Exposure to hypoxia was shown to increase ARG1 and iNOS expression in MDSCs with subsequent production of peroxynitrite and their conversion from antigen-specific to non-specific suppressors of T cell function, a phenomenon mediated by HIF1-α (85). Moreover, hypoxia up-regulates NOX2, iNOS, and ARG1 in many cell types (86). The mechanism underneath the modification of iNOS and ARG1 expression in MDSCs is based on the regulation of NF-κB. The *iNos* promoter contains also a hypoxia response enhancer (HRE) sequence, which potentiates NF-κB-dependent iNOS up-regulation in macrophages (87). Consistent data highlight the role of macrophage-derived-HIF1α in generating a localized immune privileged site within the tumor milieu via iNOS and ARG1 induction (88). Furthermore, the increased production of NO establishes a positive feedback loop on HIF1-α levels: NO-dependent impaired activity of PHD stabilizes HIF1-α and NF-κB, thus amplifying the expression of genes under their control, even under physiologic oxygen environments (89). These observations should be taken into consideration for designing new anti-angiogenic, immunotherapeutic approaches based on HIF1-α-targeting to improve the efficacy of the already available anti-VEGF therapies.

In tumor microenvironment cytokines and chemokines contribute to RNS-mediated myeloid immune suppression by multiple strategies. First of all, they can influence hematopoiesis toward the differentiation of progenitors to MDSCs and TAMs (90) and orchestrate MDSC trafficking to peripheral tissues (i.e., to the tumor) (91). Second, they can tune the expression of iNOS and ARG1. This is the case of granulocyte-macrophage colony-stimulating factor (GM-CSF) and IL-6. GM-CSF stimulates the maturation of mouse hematopoietic progenitors to DCs when used alone, and to MDSCs in combination with IL-6 (90). However, GM-CSF binding to its receptor activates different signaling cascades including JAK/STAT, mitogen-activated protein kinase MAPK, and NFκB (92) whereas IL-6 mainly induces MDSC differentiation by STAT3 activation (93). The selective induction of iNOS, ARG1, or both depends on which cytokines are involved. For example, during a pathogen infection, macrophages exclusively up-regulate iNOS to increase their microbicidial activity in response to pathogen-associated molecular patterns and a Th1 inflammatory stimulus like IFN-γ, IL-12, and TNF-α. This phenotype is mediated by NF-κB activation and binding of the IRF-1 element of the *iNos* promoter (94). Th1 cytokine release is a characteristic of classic macrophage activation and plays a fundamental role in central immunity stimulation. On the contrary, alternative macrophage activation, which promotes humoral immunity, allergic and antiparasitic responses, tissue repair, and fibrosis is linked to release of Th2 cytokines, such as IL-4, IL-10, and IL-13 cytokines, which are responsible for ARG1 up-regulation as well as iNOS down regulation (95). This phenotype is a general feature shared with DCs and granulocytes (96) and is the result of the coordinated action of PU.1 STAT6 and c/EBPβ transcription factors (97) on *Arg1* promoter. Cytokines can act also indirectly on myeloid cells modifying their sensitivity to either Th1 or Th2 stimuli. IL-23, for example, can increase Th2-mediated ARG1 induction by up-regulating IL-4-Rα and IL-13-Rα1 chains (98). Moreover, the induction and the activity of l-Arg-metabolizing enzymes are both regulated by other intracellular biochemical pathways and by competition for the same substrate, the amino acid l-Arg (99). Anti-inflammatory cytokines (i.e., TGF-β or IL-10) tune the iNOS/ARG1 balance toward the up-regulation of the latter. TGF-β is a pleiotropic cytokine that possess either inflammatory or regulatory properties depending on the cellular and environmental context (100); however, TGF-β can mediate immune suppression by indirect mechanisms, for example, increasing ARG1 activity and decreasing iNOS expression that limits macrophage-dependent cytostasis of fibrosarcoma cells (101). Interestingly, TGF-β-driven iNOS down regulation is mediated by an increase of ELK3, a transcription repressor which decreases the expression of *iNos* gene after binding to its promoter sequence (102).

There are some important exceptions in cytokine-mediated tuning of iNOS and ARG1 expression, which make the definition of either classical or alternative macrophage activation in the regulation of the two genes rather simplistic and sometimes inaccurate. In mouse macrophages, LPS, a bacterial product commonly associated with Th1 cytokine release, is able to activate both enzymes. Moreover, in tumor environment myeloid cells can restrain T cell functionality by up-regulating both enzymes. The concomitant activity of ARG1 and iNOS is a peculiar property of MDSCs, or at least of a subset of this heterogeneous population of suppressor cells induced by tumors in mice and humans (26, 103, 104). The molecular bases for the synergism might involve a double cross-talk occurring during the encounter of T cells and MDSCs. IFN-γ released by antigen-activated CD8^+^ T cells is the primary (but not unique) trigger for autocrine production of IFN-γ and IL-13 by MDSCs. These cytokines are then necessary to provide a sustained expression and activation of both iNOS and ARG1, for a sufficient time to affect T lymphocyte proliferation and survival (103). This scenario reconciles the needs of both limiting the extent of T cell suicide/inactivation only to those cells that are activated by the antigen and fully arm the suppressive program in MDSCs (103); furthermore, IFN-γ produced by activated MDSCs primes other nearby MDSCs, even though this could be limited by the necessity of a cell–cell contact with antigen-activated CD8^+^ T lymphocytes (13, 103). It is also clear that different T cell responses, biased toward either a Th1 or Th2 type cytokine production, might selectively activate either NOS- or ARG-dependent immune regulation, respectively.

## Immune Suppression Promoted by RNS in Tumor Milieu

A number of evidence indicates that peroxynitrite is toxic for lymphocytes and can prime T cells to undergo apoptotic death through different pathways involving inhibition of protein tyrosine phosphorylation via nitration of tyrosine residues (105) or by nitration of the voltage-dependent anion channel protein, a component of mitochondrial permeability transition pore (106). Nitrotyrosine, a marker of nitrative stress in tissues, is found in thymic extracts and thymic sections and co-localized with apoptotic cells, suggesting that RNS are also involved in thymic apoptosis *in vivo* (107). However, molecular explanations on how peroxynitrite released by myeloid cells can induce tolerance in antigen-specific CD8^+^ T cells were provided only recently.

Peroxynitrite interferes with peptide binding efficiency of MHC class I molecules as result of MHC class I and ligand–peptide complex nitration/nitrosylation on tumor cells; this aspect is only a part of the immunosuppressive barrier generated by MDSCs to impair tumor-specific immune responses. Neoplastic cell preconditioning with peroxynitrite *in vitro* reduces immunogenic peptide–MHC (pMHC) complex formation without affecting MHC class I expression on cell surface resulting in impaired recognition of tumor cells by CTLs. This effect was observed either for naturally processed or synthetic peptides. Peroxynitrite modifies the α chain of the MHC class I molecule only if the latter is not already loaded with an epitome peptide. This hypothesis was initially proved using artificially transduced antigen (OVA peptide) and confirmed with naturally processed melanoma antigen gp100 (108). Since nitrated proteins are mainly localized in sub-cellular compartments, like in peroxisome-containing granules, mitochondria, and endoplasmic reticulum (109), it is conceivable that the MHC class I α chain is nitrated/nitrosylated by peroxynitrite in the endoplasmic reticulum, just before assembly with β2 microglobulin and antigenic peptide. Nitration/nitrosylation can also affect immunogenic peptides, reducing their binding to MHC molecules. The conversion of tyrosine residues to nitrotyrosine on peptide epitopes could influence either TCR-contact or MHC class I and II contact positions, with profound impact on T cell responses (110–112). Elevated levels of nitrated proteins have been detected in patients with different type of cancers, i.e., bladder, colorectal, breast, and lung carcinoma, melanoma and Hodgkin lymphoma (111). In addition, in a transplantable model of mouse fibrosarcoma showing extensive myeloid cell infiltration, high levels of nitrotyrosines were found in both cytoplasm and nuclei of tumor cells (113). In the presence of RNS, such as in tumor microenvironment, the likelihood for a peptide epitope to be subjected to nitration/nitrosylation events seems thus quite high. Similarly, nitrogen-induced PTM of proteins can break the tolerance toward self-antigens and initiate autoimmune diseases. A cytochrome C-derived epitope encompassing nitrotyrosine in position 97 was shown to trigger a T cell restricted immune response against the self-protein (110). Accordingly, activated antigen presenting cells (APCs) were capable of stimulating CD4^+^ T lymphocytes with chemically modified peptides (114). Thus, from a therapeutic point of view, PTM of proteins and peptides may be exploited to break the tolerance against weak tumor-associated antigens.

Alternatively, peroxynitrite can act on the TCR side by nitrating/nitrosylating α and β subunits and the CD8 co-receptor molecule (115): *in vitro* and *in vivo* experiments confirmed the molecular modeling predictions of tyrosine residues susceptible to nitration. These modifications impair recognition of pMHC complex and promote dissociation of the CD3 ζ chain from the αβ TCR complex, thus disrupting TCR signaling (116). RNS action on T cells does not appear to be limited to nitration/nitrosylation of the crucial molecules α/β TCR, CD3, and CD8 but it also involves a TCR ligand-independent, transient tyrosine phosphorylation of the CD3 ζ chain, sustained for about 24 h, which delivers an abortive signal preventing further T cell activation by TCR ligands (117). In addition, a rapid down regulation of CD4 and CD8 co-receptors was observed following T cell exposure to RNS (117). In peripheral lymphoid organs, all the inhibitory effects promoted by MDSC-secreted RNS are primarily directed toward naïve T cells bearing the TCRs recognizing the pMHC complexes presented by MDSCs. Some experiments indicate that MDSCs are able to present tumor antigens to specific CD8^+^ T cells and in this context they should facilitate exposure of TCR to the action of the peroxynitrite they secrete. Failure to respond to stimulation with a specific peptide was observed only for CD8^+^ T cells nitrated at TCR/CD8 complex. T cells recognizing pMHC complexes different from those expressed on MDSC surface showed less TCR/CD8 nitration and retained the ability to respond to antigens presented by classic APCs. These data indicate that T cells with high affinity TCRs for pMHC complexes presented by MDSCs are more prone to peroxynitrite-dependent inhibition by MDSCs (115, 116).

Another RNS-promoted mechanism of immune suppression relies on the modification of chemokines. As a result of RNS exposure, these molecules might undergo PTM that alter their chemotactic properties, impairing the recruitment of T cells and keeping them at the periphery of the tumor lesion, unable to reach the tumor core and exert their cytotoxic action (118). Chemokines are organized in a complex and dynamic network, which coordinates leukocyte trafficking both under physiological and pathological conditions. To perform their action these proteins bind to specific receptors associated to heterotrimeric G proteins, which are present heterogeneously on all leukocytes (119–121). Receptor activation leads to the triggering of signaling pathways including inositol-phosphate 3-kinase (IP3K) and NF-κB transcription factor. The signaling cascade activates a series of events ending in a rapid remodeling of the cytoskeleton, with increase in integrins and adhesion molecules that promote adhesion, extravasation, and leukocyte migration to the target sites (119). Normal chemokines activity is also regulated by PTM such as proteolytic processing, glycosylation, and deamination (122, 123). RNS inhibit migration of T lymphocytes induced by some chemokines, such as CXCL12, CCL21, CCL2, and CCL5. RNS can down-regulate the expression of CXCR4 on T cells through alteration of the TCR/CD3 signaling complex and reduce CXCL12–induced T cell migration (124). When exposed to the action of RNS, mouse and human CCL2 become nitrated at the level of tyrosine and tryptophan residues respectively; CXCL12, is subjected to tyrosine nitration as well. This PTM reduces the binding affinity of CCL2 to its receptor CCR2, affecting drastically tumor homing of CD8^+^ T lymphocytes. Nitrotyrosines are found in tissue sections of hyperplastic human lymph nodes obtained from surgical resections of patients with lung, colon, and prostate cancer (105, 124). Surprisingly, the nitrated/nitrosylated CCL2 does not lose the ability to recruit MDSCs to the tumor, likely due to higher expression of CCR2 in myeloid than in T cells (118).

## Available Technologies and New Approaches for Identification of Nitrogen-Induced PTM

Nitrogen-induced PTM is a process promoted by oxidative environments in presence of NO and associated with a number of different diseases such as autoimmunity (125), neurodegenerative disorders (126), and cancer (118). Exactly like other PTM (i.e., phosphorylation/dephosphorylation), RNS action on target proteins modifies their biochemical properties. Indeed 3-nitrotyrosine, the most common and studied RNS-derived PTM, shows peculiar chemical characteristics, which makes it different from normal tyrosine (127) and can modify the protein tertiary and quaternary structure, and therefore its activity. Given the clinical relevance of PTM and 3-nitrotyrosine, many efforts have been done in designing and developing tools to identify which proteins are subjected to NO modification, the position of the chemical alteration, and its biological role. Many of them are based on the use of chromatography [i.e., HPLC: Ref. (128)] or mass spectrometry (129), or both (130). These techniques are also reviewed elsewhere (131). However, a major issue in defining the “nitrome” is related to the very low representation of nitrogen-induced PTM within the microenvironment. Indeed, only few tyrosines are modified *in vivo* under inflammatory conditions: in this context the specificity and resolution of the detection technologies may represent a limiting factor (132). Thus, enrichment of nitrogen-induced PTM (such as with antibodies that specifically bind modified proteins) represents an important technological improvement. Accordingly, the development of 3-nitrotyrosine-specific antibodies opened the way to a body of publications focused on semi-quantitative analysis of 3-nitrotyrosine in complex biological samples. Anti-3-nitrotyrosine antibody improved already available methods for the identification of proteins subjected to nitrogen-induced PTM in complex samples separated through gel-based approaches. Indeed, a semi-quantitative evaluation of 3-nitrotyrosine levels in complex samples can be performed by 2D protein separation followed by transfer on filter and blot with anti-3-nitrotyrosine antibody; furthermore the alignment of the blot with 2D gel identifies the spots that can be excised for further protein identification by MS. However, the strength of this enriching technique strictly relies on the antibody specifications (i.e., affinity). Moreover, some PTM may be ignored depending on the tridimensional availability of the 3-nitrotyrosine residues to the antibody. Recent works provide an example of how these innovative detection tools can be exploited to characterize the biological impact of nitrogen-modified proteins involved in regulating peripheral immune response. Through the implementation of liquid chromatography and MS, in fact, mouse and human CCL2 were found to be nitrated on tyrosine and tryptophan residues upon exposure to RNS, respectively. To confirm the biological impact of nitrogen-induced CCL2 modification, a single-domain recombinant antibody was isolated that selectively binds modified CCL2 and can be used for its detection (118).

Tyrosine is the preferential target of RNS attack; however, a growing body of evidence highlights PTM of other aromatic amino acids, such as tryptophan, which can alter protein function and cellular metabolism as well. Peroxynitrite and oxidant environments modify human Cu, Zn-superoxide dismutase and bovine serum albumin at tryptophan residues (133). Interestingly, these modifications were associated with a 30% decrease in SOD enzymatic activity. Moreover, RNS rich environments promote a broader profile of chemical alterations than those observed on tyrosines. Among them, 1-*N*-nitrosotryptophan and 6-nitrotryptophan (6-NO_2_Trp) are the most representative products after peroxynitrite exposure (133). However, the modest presence of tryptophan nitration residues, compared with tyrosine, represents a limiting factor in the identification of proteins that undergo RNS-dependent PTM. This is the result of the lower tryptophan frequency in the primary protein structure, lower exposition to the solvent (hydrophobicity), and higher redox potential than tyrosine. Despite of this technical issue, the identification of nitrotryptophan-modified proteins represents a key task since solvent exposed tryptophan usually participates in molecule interactions. Indeed, the low amount of modified tryptophan does not allow predicting the biological impact that its alteration will cause. A recently developed antibody, which selectively binds 6-NO_2_Trp, was successfully used in western blot to detect and identify nitrotryptophan-containing proteins in peroxynitrite exposed rat pheochromocytoma cells (134). The access to reagents, which specifically label nitrotryptophan and other nitrogen-dependent modifications, will increase the body of data on nitrogen-dependent PTM and provide a detailed snapshot of the post-translational signature induced by nitrogen under physiologic and pathologic contexts.

## Clinical Relevance of RNS

The detection of the nitroxidative stress signature in various human tumors and stages may provide information about its role in tumor formation and progression. The correlation of nitration level with clinical parameters can additionally offer prognostic tools and allow the development of therapeutic strategies.

Expression and distribution of iNOS have been evaluated both at the mRNA and protein level in many tumors and conflicting results of these analyses have been reported (135, 136). Over expression of iNOS is frequent in various human tumors but a correlation with tumor stage, grade, or metastases, as well as with poor prognosis is reported only in certain types of cancer. In gastric cancer (137) and melanoma (138), for example, iNOS level could be a predicting biomarker for poor prognosis. Anyway, other types of cancer show either controversial results such as in colorectal, breast, brain, lung, and cervical cancer (139, 140) or no correlation at all, as in bladder carcinoma, pancreatic and cervical cancer (141). Regarding the other two NOS isoforms, NOS3 expression strongly correlates with iNOS presence in breast carcinoma (142) and NOS1 has been detected in some oligodendrogliomas and neuroblastomas cell lines (143). However, detecting the presence of NOS protein is only an indirect method to evaluate nitroxidative stress (144). In this respect, more accurate information is provided by staining with anti-nitrotyrosine antibodies (145). Table 1 compares nitrotyrosine staining pattern identified in different human tumors. NOS and ARG detection are shown as well, as they are involved both in a synergistic peroxynitrites generation and in independent immunosuppressive activities (26, 146). Immunohistochemistry (IHC) confirms that many tumors generally display higher nitration level than control tissues. Compared to other quantitative approaches that finely measure PTM levels, IHC can provide the localization of the cellular type and compartment subject to nitration: nitrotyrosine staining is usually localized in neoplastic cells, but it is also observed at the level of stromal cells as reported for colon, gastric, breast cancer, and mesothelioma (147–150). Moreover, nitrotyrosine staining is mostly diffused in the cytoplasm; additional focal nuclear pattern of staining is described for breast, colon, gastric, and papillary thyroid cancer (147–149, 151). The presence of the positive staining, however, highlights the localization of tyrosine-nitrated proteins but it does not provide any information about the source of peroxynitrite that generates this modifications. Indeed, RNS can easily diffuse out of the membrane and accomplish their function in a different cellular target.

**Table 1:** **Nitrotyrosine presence in human cancers**.

Type of cancer	Nitrotyrosine staining pattern	Clinical and/or experimental correlation	NOS/ARG isoforms	Reference
Pancreatic adenocarcinoma	Increased staining for iNOS and nitrotyrosine in the ductal epithelium of pancreatic tumor tissue	Enhanced expression of FGF-1/2 and FGF receptors associated with an environment of oxidative stress. Tyrosine-nitrated c-Src found only in patient tissues	iNOS	(152)
Prostate cancer	Increased staining for nitrotyrosine in the epithelial cancer cells, some hot spots in the TILs	Dysfunctional *in situ* TIL responses	iNOS/ARG2	(124)
Glioblastoma	Increased staining for nitrotyrosine in glioma cells and possibly in passenger leukocytes	Inhibition of wild-type p53 function in malignant glioma cells	NOS1/iNOS	(143, 153, 154)
Lung squamous cell carcinoma and well differentiated adenocarcinoma	Increased staining for nitrotyrosine in tumor cells but not in adjacent normal areas	Nitration of metabolic enzymes, structural proteins, and proteins involved in prevention of oxidative damage	NOS3/iNOS	(155)
Head and neck squamous cell carcinoma	Increased staining for nitrotyrosine in tumor cells	Increased nitrotyrosine staining in the progression of oral mucosa from normal to dysplastic and neoplastic changes	NOS3 and iNOS	(47, 156)
Mesothelioma	Diffuse and variable cytoplasmic nitrotyrosine staining for both cancer and stromal cells	More intense nitrotyrosine staining in mesotheliomas with higher mitochondrial manganese superoxide dismutase (MnSOD) expression	ND	(150)
Colon cancer	Cytoplasmic and nuclear nitrotyrosine staining in cancer and stromal cells	Reduction of PHA-dependent proliferation of human T lymphocytes in the presence of supernatant from a culture of cytokine-induced, NO-producing colon carcinoma cells	iNOS	(147, 157)
Breast cancer	Diffuse cytoplasmic and focal nuclear nitrotyrosine staining for cancer cells, stromal macrophages and neutrophils	Correlation between high nitrotyrosine staining and VEGF-C immunoreactivity and lymph node metastasis; nitrotyrosine staining proposed as an independent prognostic value for overall survival	ND	(149)
Melanoma	Weak to strong diffuse and paranuclear staining of melanoma cells	Strong correlation between poor survival with iNOS and nitrotyrosine expression by melanoma cells in patients with stage III disease	iNOS	(138)
Papillary thyroid carcinoma (PTC)	Weak to high-grade staining in the cytoplasm and focally in the nucleus in PTC cells	High-grade nitrotyrosine staining was correlated with VEGF-D immunoreactivity and lymph node metastasis	iNOS	(151, 158)
Bladder carcinoma	Nitrotyrosine staining mainly in endothelial cells and certain stromal cells of malignant bladder tumors; weak staining in tumor cells	ND	iNOS/NOS3	(159)
Gastric adenocarcinoma	Cytoplasmic and nuclear nitrotyrosine staining in tumor and adjacent stromal cells	Expression of iNOS and nitrotyrosine with a high AI is associated with a poor survival; correlation of both iNOS and nitrotyrosine with cancer subtype (prevalence in tubular carcinoma) but no significance correlation with clinical parameters	iNOS	(148, 160)
Renal cancer	Nitrotyrosine staining mainly in tumor cells, but occasionally also in stroma and in the tubular cells of non-neoplastic renal tissue	Nitrotyrosine associated with high-grade tumors	ND	(161)
Gynecological cancer	Moderate nitrotyrosine cytoplasmic staining in most of invasive ovarian carcinomas	In invasive ovarian carcinomas there is no association with any clinico-pathological parameters, whereas it was observed a strong correlation with expression of the antioxidant enzymes peroxiredoxin IV and thioredoxin	iNOS	(162, 163)

*ND, not determined; 8-OHdG, 8-hydroxydeoxyguanosine; VEGF, vascular endothelial growth factor; MnSOD, mitochondrial manganese superoxide dismutase; TILs, tumor-infiltrating lymphocytes; AI, apoptotic index*.

Besides qualitative observations, correlations of nitrotyrosine staining with clinical parameters are described only for certain tumor types and positivity is generally associated with worse outcome. In head and neck squamous cell carcinoma, the staining increases with tumor progression, suggesting a possible role of peroxynitrite generation during carcinogenesis (47). In advanced melanomas, a strong correlation exists between poor survival, iNOS expression, and nitrotyrosine presence (138), while in a cohort of patients with invasive breast cancer, nitrotyrosine staining correlates with lymph node metastasis and it may serve as a significant prognostic factor for long-term survival since high nitrotyrosine levels are related with poor overall survival (149). Literature for gastric adenocarcinoma describes heterogeneous and controversial findings: one cohort of patients exhibited a difference in nitrotyrosine staining according to the tumor type, with tubular carcinoma subtype showing the higher positivity (160). In another study, a correlation was described between nitrotyrosine and tumor apoptosis showing that patients with high apoptotic index, elevated iNOS expression, and nitrotyrosine had a poor survival (148). Several factors can potentially explain tumor differences regarding the correlation between RNS and clinical evidences. In first instance, each tumor type can be subjected to a peculiar nitrosative stress level depending on either the abundance or type of RNS-producing cells. Moreover, tumor cells may be more or less resistant to oxidative environments depending on the activity of detoxifying mechanisms such as autophagy and availability of molecules and proteins with scavenging properties. Thus, nitrotyrosine staining is an inflammation indicator that provides useful information on the clinical outcome when used in combination with other parameters such as the immunological context (T cell and myeloid infiltrate, M1/M2 macrophage levels) and local microenvironment (Th1/Th2 cytokines for instance). In addition to clinical parameters, there are few published works describing an association between nitrotyrosine positivity with other biological factors linked to tumor progression. In a cohort of pancreatic adenocarcinoma patients, in fact, the tumor environment was characterized by oxidative stress linked to the expression of mitogen fibroblast growth factor (FGF)-1/2 and their receptors, which all play a role in pancreatic cancer development (164). In a group of advanced breast cancer patients, nitrotyrosine positive staining is associated with the presence of the angiogenic factor VEGF-C (149). In prostatic cancer, NO and peroxynitrites promote tumor growth through the suppression of anti-tumor immune response. Accordingly tumor-infiltrating lymphocytes (TILs) isolated from prostate cancer by using collagen gel matrix-supported organ cultures were unresponsive to mitogen stimuli; *ex vivo* prostate neoplastic cells generate peroxynitrite responsible for tyrosine nitration in TILs and blockade of peroxynitrite production restored TIL responsiveness (124). Similar dysfunction in T cell proliferation when exposed to NO-producing tumor cell line supernatants was described in colon carcinoma (157).

## RNS: Therapeutic Implications

To date, the nitrotyrosine detection in human cancer tissues does not have a uniform relevance with respect to predicting clinical disease progression in cancer patients. The reasons of this lack of homogeneity are not known and might depend, in part, on the limits in the available tools to detect nitrogen-induced PTM and the dichotomic role played by NO (165). Specific locations and targets of tyrosine nitration need to be clearly identified in order to understand in full the biological impact of PTM and find potential candidates for new therapeutic approaches. In glioblastoma multiforme (GBM), for example, the key tumor suppressor protein p53 exhibits peroxynitrite-mediated modifications *in vivo* and, moreover, malignant glioma cell lines treated with peroxynitrite show wild-type p53 function inhibition (153, 154). Strategies designed to target RNS and restore p53 functionality in tumor cells are thus one example of a promising field of research with translational outcomes.

As discussed above, the l-Arg metabolizing enzymes NOS and ARG are key enzymes for the nitrogen-induced immunosuppressive properties of MDSCs and TAMs. In an *in vitro* model of human prostate carcinoma, only the contemporary inhibition of ARG and NOS with NG-hydroxyl-l-arginine (NOHA) and l-NG-monomethyl arginine citrate (l-NMMA) respectively, was effective in reducing intratumoral nitrotyrosine staining and this directly correlated with the ability in restoring TIL responsiveness to various stimuli (124). In untreated cultures, in fact, prostate TILs did not react either to antigens or signals bypassing TCR binding, indicating profound state of immune dormancy/anergy (124). Overall, mouse and human studies have clearly recommended that drugs aimed at reverting T lymphocyte unresponsiveness in tumor-bearing hosts should target both ARG and NOS. The pharmacological approach manipulating l-Arg metabolism via ARG1 and iNOS have shown therapeutic potential in preclinical studies, suggesting its use as adjuvant in combination with treatments that directly enhance the immune response. NO-donating drugs such as nitroaspirin (classic aspirin molecule covalently linked to a NO donor group) inhibit iNOS through various mechanisms (166, 167). MDSC-derived immune suppression could be relieved either by nitroaspirin, the combination of ARG and NOS inhibitors, or peroxynitrite scavengers, whereas simple NO-donors or nitroaspirin analogs lacking the NO-donating group were ineffective (168). The salicylic portion (with the attached spacer) of nitroaspirin was necessary for ARG1 blockade whereas NO donation was important for the feedback inhibition of iNOS (166, 167). *In vivo* administration of nitroaspirin resulted in decreased intratumoral staining with anti-nitrotyrosine and anti-iNOS antibodies, together with a reduced intratumoral ARG activity (168). Despite these positive results, nitroaspirin was not effective as an adjuvant for adoptive cell transfer therapies (ACT; unpublished data). On the contrary, a member of a new class of NO-donating drugs, AT38 ([3-(amino carbonyl) furoxan-4-yl]methyl salicylate), was shown to increase adoptive cell therapy effects. This compound is able to revert RNS-induced PTM of chemokines necessary for T cell tumor invasion (118) and to reduce the *in vivo* generation of peroxynitrite that can impair T cell migration to the tumor. Interestingly, RNS targeting might represent a new innovative therapeutic strategy for a broad spectrum of RNS-induced diseases, like neurodegenerative and cardiovascular one. AT38 efficacy, in fact, is not limited to the tumor microenvironment but can be useful more in general within an inflammation context. In a mouse model of epicutaneous vaccinia virus infection, AT38 treatment restored CD8^+^ T cell relocation to epidermal foci, suggesting that peroxynitrite production control CD8^+^ T cell entry, and consequent activity, into viral lesions (169).

As described above, intratumoral ARG activity can be sustained by other factors, such as COX, acting on tumor-infiltrating myeloid cells. In several preclinical trials the use of COX2 inhibitors, such as celecoxib, down-regulated ARG1 expression, delayed tumor growth, and stimulated anti-tumor response (81, 170–172). In a mouse model of mesothelioma, Celecoxib improved survival when used in combination with DC-based immunotherapy (172). Moreover, inhibition of ARG1 and iNOS activity in MDSCs can be achieved by phosphodiesterase-5 (PDE5) inhibitors (sildenafil, tadalafil, and vardenafil), which can restore the spontaneous anti-tumor T cell response (173). *N*(6)-(1-iminoethyl)-l-lysine-dihydrochloride (l-nil) is an iNOS-selective small molecule antagonist that showed potent anti-proliferative properties on melanoma cells increasing survival in tumor-bearing mice in combination with chemotherapy (174). To improve cellular antioxidant properties, thus limiting ROS and RNS generation, a group of anti-inflammatory molecules can represent an alternative option: they are synthetic triterpenoids that activate a transcription factor regulating antioxidant genes, the nuclear factor (erythroid-derived 2)-like 2. The synthetic triterpenoid CDDOMe reduced ROS and peroxynitrite levels in MDSCs and abrogated MDSC-dependent immune suppression both in several tumor mouse models and in pancreatic cancer patient; however, ARG activity or NO levels were not affected in MDSCs (175). It is thus possible to exploit in current therapeutic protocols, the association of different drugs affecting either concurrent or alternative steps in the biochemical production of peroxynitrite. Other antioxidant compounds have been validated *in vitro* and are currently investigated as candidates for cancer therapy. For instance, 3,4′,5-trihydroxy-trans-stilbene (resveratrol), a polyphenolic natural product described as free radical scavenger was able to induce apoptosis in several cell lines by regulating proteins that patrol cell cycle such as p53 and cyclin dependent kinases (176–178). Accordingly, diferuloylmethane (curcumin) showed similar properties *in vitro*. This phenolic compound can be considered more than just an oxidant scavenger molecule since it inhibits the NF-κB activation reducing iNOS and DNA-dependent protein kinase catalytic subunit levels; additionally curcumin induces the up-regulation of p21, p27, p53, and the checkpoint kinase 2 expression, thus promoting the G2/M cycle arrest and apoptosis in human melanoma cell lines (179). Given their similar biological properties, resveratrol and curcumin have been tested together in different set-ups with promising results; their combination was able to reduce the proliferation of Hepa 1–6 hepatocellular tumor cells *in vitro* (180) and of HCT-116 colon cancer cells, *in vivo* (181).

As described above, nitrosative stress plays a role in regulating autophagy in MCF-7 breast tumor cells (55), therefore therapeutic approaches targeting this pathway can be also considered. Autophagy, indeed, is a double-edged sword, which can either promote or control tumor progression; when this process is impaired, cells become more sensitive to RNS and oxidative conditions resulting in higher accumulation of genetic alterations, which increase both apoptosis and neoplastic evolution. From the other side sustained autophagy progressively depletes cells from proteins, membranes, and organelles until death. Therefore this process can be exploited to promote tumor apoptosis by using autophagy activators, such as rapamycin (54).

In conclusion, based on published data, many agents have shown potential benefits in combination with immunotherapy in preclinical studies. l-Arg metabolism manipulation via ARG and NOS can contribute in the regulation of tumor-infiltrating immune cells, reducing MDSC accumulation and promoting T cell infiltration (81, 118, 168, 170), thus representing a potential adjuvant for immune therapeutic approaches. However, before reaching the clinic, further knowledge is necessary, such as the evaluation of systemic side effects and the assessment of cancer types that would receive a real benefit after modulation of l-Arg metabolism.

**Figure 2:**
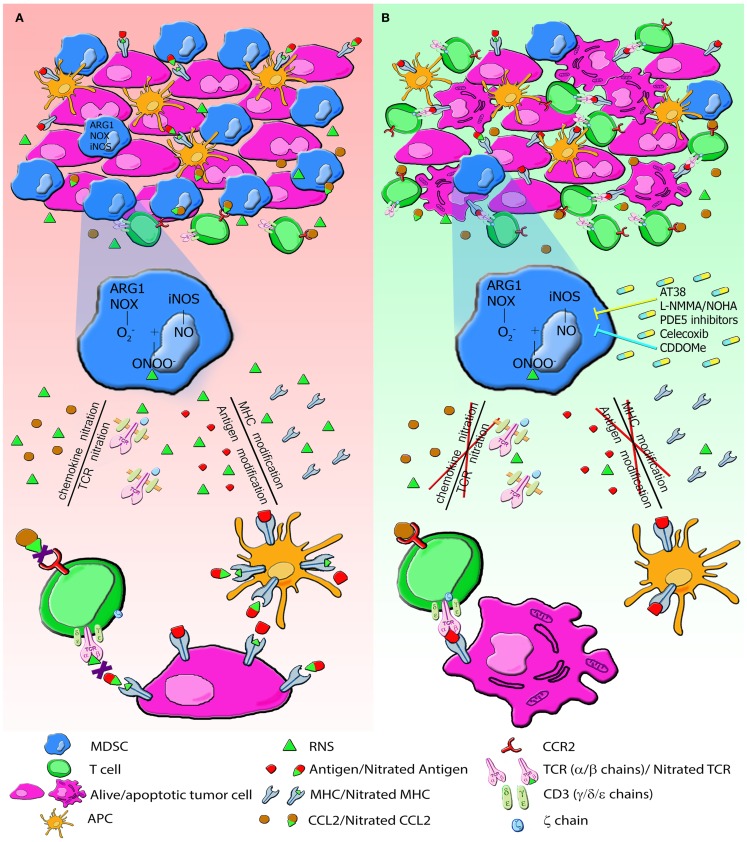
**The generation of RNS in tumor environment induces PTM that alter immune responses to tumors**. **(A)** MDSC and tumor cells express high level of NOX, ARG1, iNOS and produce large amount of RNS. RNS induce nitration/nitrosylation of either MHC class I molecules or peptide ligands on tumor cells and TCR chains on T lymphocytes, thus resulting in impaired recognition of tumor cells. RNS also modify chemotactic proprieties of chemokines. CCL2 nitration reduces the binding affinity to its receptor, CCR2, drastically affecting tumor homing of CD8^+^ T lymphocytes. All these modifications alter the recruitment of T cells and their cytotoxic function. **(B)** Administration of molecules AT38, l-NMMA, PDE5, Celecoxib, and CDDOMe through the enzymatic inhibition of NOX, ARG and NOS blocks nitration/nitrosylation, restores immune functions, and increases lymphocyte infiltration.

Figure 2 summarizes MDSC activity in tumor environment (Figure 2A) and the effects of the above mentioned inhibitory compounds (Figure 2B).

## Conclusion

Reactive nitrogen species-induced modifications of proteins play a critical role in distracting the immune system from recognizing and eliminating tumor cells, and most likely act as an important barrier that limits the effectiveness of immunotherapy approaches. Targeting RNS can substantially improve the effect of cancer immune therapy and also their detection could be used as biomarker to define the prognosis and evaluate the clinical outcome of patients. In many types of cancer, tumor-infiltrating myeloid cells, such as MDSCs and TAMs, act as the major source of RNS in tumor tissues. Therefore, their elimination or functional block could also contribute to a fundamental reduction in RNS. Although several mechanisms of RNS-mediated effects on the immune system in cancer have been suggested, the underlying molecular processes need further elucidation. In this context, the development of new tools for the identification of nitrogen-induced PTM will allow clinicians and researchers to provide a signature of nitrated proteins, impaired signaling pathways, and cellular dysfunctions activated by those modifications under specific pathologic conditions, opening the way to a more customized therapeutic intervention. New cancer treatment protocols include combinatorial approaches such as tumor microenvironment preconditioning to eliminate patient immune dormancy. In this scenario, targeting RNS-induced PTM represents a new emerging frontier of combinatorial cancer therapy that can improve the efficacy of either active or passive immunotherapy.

## Conflict of Interest Statement

The authors declare that the research was conducted in the absence of any commercial or financial relationships that could be construed as a potential conflict of interest.
